# An interview with Giorgio Fiorelli

**DOI:** 10.1590/2177-6709.23.5.024-038.int

**Published:** 2018

**Authors:** Giorgio Fiorelli, Gidalti Bueno Linhares, Maurício Tatsuei Sakima, Renato Parsekian Martins, Wislei de Oliveira

**Affiliations:** 1 » Born in Arezzo, Italy, 1958. » Medical degree, dental degree and orthodontic postgraduate program at the University of Siena. » Private practice focused in Orthodontics since 1983. Currently in Arezzo, Florence (Italy) and Richterswil (Zurich, Switzerland). » Part time assistant Professor at the orthodontic department of the University of Siena from 1993, where he has been responsible for adult orthodontic treatments and teaching of biomechanics. » Affiliated to the orthodontic department of University of Aarhus, Denmark, where he has been involved in the short term and postgraduate teaching, since 1992. » Affiliated to the orthodontic department of the University at Buffalo (USA), where he has been teaching from 2011 to 2014. » Secretary General of the Italian Orthodontic society in the years 1998-99. Vice-president of Italian Society of Orthodontic Biomechanics in the years 1999-2002. » He has published about 30 papers on the above-mentioned fields. » Has co-published with Prof. Birte Melsen the “Biomechanics in Orthodontics” multimedia software, presently at release 4. » Co-writer in the book “Adult Orthodontics” edited by Birte Melsen, and in the book “Orthodontic Pearls”, edited by Larry White. » Author of the chapter “Statically determined appliances and creative mechanics” in the textbook “The biomechanical foundation of clinical orthodontics”, edited by Prof. Burstone and Choy. » Responsible for “Adult orthodontics” postgraduate program at the University of Siena, from 2002 to 2013. Visiting professor at University at Buffalo, from 2012 to 2015. » Currently holds private courses of biomechanics in Italy, Poland and Portugal, and is head of the International Orthodontic Biomechanics School, which organizes an intensive five-week program attended by orthodontists coming from countries in all continents.; 2 » DDS, Universidade Estadual de Ponta Grossa (Ponta Grossa/PR, Brazil). » Specialist in Orthodontics, ABO-PR, Escola de Aperfeiçoamento Profissional (Guarapuava/PR, Brazil). » Founding member of BIOMEDE Biomechanics Development - International Association for Development and Spread of Orthodontic Biomechanics Knowledge. » Postgraduate in Orthodontic Biomechanics, IOSS - GmbH, International Orthodontics School & Services (Wollerau, Switzerland).; 3 » Master and Doctor in Orthodontics, UNESP, FOAr (Araraquara/SP, Brazil). » Postdoctoral degree in Orthodontics, Aarhus University, Royal Dental College (Aarhus, Denmark). » Assistant Professor, UNESP, FOAr, Departamento de Clínica Infantil (Araraquara/SP, Brazil). » Guest Lecturer, Aarhus University, Royal Dental College, Postgraduate programme in Orthodontics (Aarhus, Dinamarca) » Coordinator, APCD, FAOA, Curso de Especialização em Ortodontia (Araraquara/SP, Brazil).; 4 » Master, Doctor and Post-doctoral degree in Orthodontics, UNESP, FOAr (Araraquara/SP, Brazil). » PhD sandwich in Orthodontics, Texas A&M School of Dentistry (Dallas, USA). » Assistant Professor, UNESP, Pós-graduação em Ciências Odontológicas (Ortodontia) (Araraquara/SP, Brazil). » Assistant Editor of Dental Press Journal of Orthodontics and Revista Clínica de Ortodontia Dental Press. » Author of the column “Biomechanics”, Revista Clínica de Ortodontia Dental Press.; 5 » Specialist in Orthodontics and Facial Orthopedics, USP, FOB, FUNBEO (Bauru/SP, Brazil.) » Postgraduate in Orthodontic Biomechanics, IOSS - GmbH, International Orthodontics School & Services (Wollerau, Switzerland). » International member of the American Association of Orthodontists (AAO).

**Figure:**
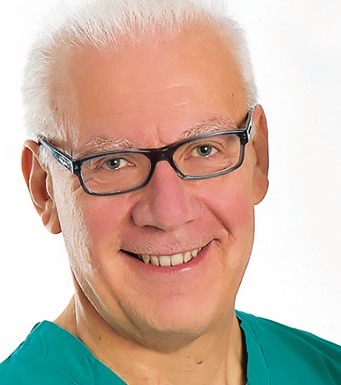

I am extremely honored and happy to coordinate the interview of one of the greatest names in European Orthodontics: Giorgio Fiorelli. In 2014, I was at a moment in my life that I like to describe as a professional limbo, and, without motivation to continue in Orthodontics, was considering “getting out” and trying a new career out of Brazil. Destiny, though, brought me the opportunity to meet Dr. Giorgio Fiorelli and Dr. Birte Melsen during the Orthodontic Biomechanics Summer School, which happened in a place in the Italian coast, in the Toscana region. Those were intense 35 days of pure Orthodontics, many calculations, many classes, eight hours a day of immersion in a world that had so far been little explored by me: Orthodontic Biomechanics. Spending time for so many days with Dr. Giorgio Fiorelli rekindled my passion for Orthodontics and showed me, once again, why I had chosen Orthodontics as my professional area. Giorgio had the opportunity to study in the Aarhus University, in Denmark, and to be a student and later, as a professor, a colleague of Dr. Birte Melsen. Giorgio is passionate about technology and software programming and development, but his greatest passion is Orthodontics. While studying and living close to Giorgio, you can see in his eyes how much in love he is for these areas of study. Therefore, he sought opportunities to bring together his two passions during his professional career. Giorgio Fiorelli developed a digital occlusogram tool and software to analyze tooth movement, and he is the main author of an e-book in Orthodontic Biomechanics, which is updated daily. Author and co-author of innumerable studies in the area of Orthodontic Biomechanics, he currently carries on research on mandibular repositioning prior to orthodontic treatment. A polyglot, Giorgio speaks English, Italian, German and French fluently and likes to play the guitar in his free time, but he confessed that currently the few hours away from Orthodontics are “spent” with his wife and children. I hope this interview rekindles the flame of love for good Orthodontics in all our readers. I thank Dental Press Journal of Orthodontics for the opportunity to coordinate an interview in this publication, one of the main journals of Orthodontics in the world. Enjoy your reading!

Sinto-me extremamente honrado e feliz em poder coordenar a entrevista de um dos maiores nomes da Ortodontia europeia: Giorgio Fiorelli. No ano de 2014, eu me encontrava em um momento que gosto de descrever como limbo profissional: sem motivação para continuar na Ortodontia, estava disposto a “jogar tudo para o alto” e tentar uma nova carreira profissional fora do Brasil. Porém, quis o destino que eu tivesse a oportunidade de conhecer o Dr. Giorgio Fiorelli e a Dra. Birte Melsen durante a *Orthodontic Biomechanics Summer School,* na Itália. Foram 35 dias intensos de Ortodontia pura, oito horas diárias imerso em um mundo, até então, pouco explorado por mim: a Biomecânica ortodôntica. Passar tantos dias com o Dr. Giorgio reacendeu minha paixão pela Ortodontia e me mostrou, mais uma vez, porque eu tinha escolhido-a como minha área profissional. Giorgio estudou na *Aarhus University*, na Dinamarca, onde foi aluno e depois, como professor, colega da Dra. Birte Melsen. Ele é um apaixonado por tecnologia e por programação/desenvolvimento de *softwares*, mas sua maior paixão é a Ortodontia. Ao estudar e conviver com Giorgio, você pode perceber nos seus olhos o quão apaixonado ele é por essas áreas; assim sendo, ele procurou unir essas duas paixões durante sua carreira profissional. Giorgio Fiorelli desenvolveu um oclusograma digital e um *software* para análise do movimento dentário, e é autor principal de um *e-book* de biomecânica ortodôntica que é atualizado diariamente. Autor e coautor de inúmeros artigos na área de biomecânica ortodôntica, atualmente ele desenvolve uma linha de pesquisa sobre o reposicionamento mandibular antes do tratamento ortodôntico. Poliglota, ele fala com fluência inglês, italiano, alemão e francês; gosta de tocar violão em suas horas vagas, mas confessou-me que atualmente as poucas horas longe da Ortodontia são “gastas” com sua esposa e seus filhos. Espero que essa entrevista reacenda a chama do amor pela boa Ortodontia em todos os leitores. Agradeço ao *Dental Press Journal of Orthodontics* pela oportunidade de coordenar uma entrevista nessa que é uma das principais revistas de Ortodontia do mundo. Boa leitura!

Gidalti Bueno Linhares (interview coordinator)

**1) Nowadays in the context of modern Orthodontics, with the advent of the self-ligating brackets, orthodontic aligners and the skeletal anchorage, how the knowledge of orthodontic biomechanics and the segmented arch technique can contribute? Maurício Sakima**

As a general principle, I believe that biomechanics will always play an essential role in orthodontics. At least until we will move the teeth by applying mechanical stress. Think to the aligner developments: the dramatic improvements that Aligntech had in the last years are mostly due to the work of an engineer who has a great experience in biomechanics, having worked beside Charles Burstone for many years: John Morton. 

The real question is another one: which should be the role of the clinician? Should we be aware of the deep mechanisms by which the appl­iances move the teeth? Or should we just press a button and let the appliance work while we watch the show? Maybe in the future, the technology of the orthodontic appliance will be so good that we will not need clinical orthodontists anymore. Now I believe we are very far from this possibility. Many orthodontic treatments require a great skill and control by the clinician and cannot be treated safely with the “automatic pilot”, this is also due to the fact that we are called to treat also more challenging and complex cases. Soon I believe that there will be two types of treatments: those that will need no orthodontist at all (a general dentist might be even overqualified), and those cases, always more complex and challenging, demanding for excellent expertise and skill. I recommend to the young orthodontists to understand that if they entrust totally the treatment to a ready-made appliance, they might have an easy life today in many cases, but not in all, and they could become jobless in the future, since this level of competence can be easily replaced by a general dentist, or even by a salesperson.

In this context, I believe that the biomechanics knowledge will be the most important asset for an orthodontist to stand out of the line. 

Regarding the segmented arch, it is an orthodontic approach that makes the orthodontist free of the boundaries of continuous archwires. It is the only technique in which we can obtain a force-driven displacement, and this is because in many situations the orthodontist is able to asses exactly the applied force system. This implies larger possibilities but also requires more knowledge. By a theoretical point of view, the segmented arch simplifies the orthodontic mechanics. I can’t imagine anything more complex to understand than the force systems generated by an initial alignment NiTi wire.^1^ ^,^ ^2^ We all use it because it is easy to apply, but it is not easy to understand and predict its effect, even if we have a good biomechanics knowledge. So, if we were all limited to use only a continuous archwire the differences between orthodontists with great or poor biomechanics knowledge would be limited. On the contrary, no one can afford to use segmented mechanics without a solid biomechanics knowledge. 

**2) What keeps us today from having a software that can precisely predict tooth movement on a continuous arch or on a segmented arch approach? Renato Parsekian Martins**

This question is not a very easy one to answer to. I will do my best, but first, let me tell you that I believe that we should rather refer to shape-driven, or force-driven mechanics rather than continuous arch or segmented arch approach. This was a concept that we have heard many years ago from C. Burstone.

For what concerns shape-driven mechanics, I believe that there are now many software and systems that have been developed and that can predict, within the inherent limits of shape-driven systems, the appliance effect very nicely. For instance, those developed by Suresmile^®^, Insigna™, Incognito™, WIN, Invisalign^®^. They all lead to a quite satisfactory “alignment”, which is what any shape-driven system can reach. We call this indiscriminate alignment: it means we get a desired relation between the teeth in one dental arch, but we have little or no control on the absolute position of the teeth within the orofacial structures and on the path that every single unit will follow to reach the alignment.

For what concerns the force-driven systems, which are the field of my own interest, things are very different. The problem here is conceptually very simple: I need to ask myself if I know the mechanical system that I produce and if I know the biological reaction to it.

Now, do we know the 3D force system we apply on every tooth? If you work with a continuous archwire, the answer is an absolute no and there is no chance soon to have the possibility of having this information at the clinical level. This cancels any hope to predict the exact movements that every unit will perform. I believe that a continuous arch approach should be used only to obtain limited amounts of dental movements. In this case, any mistake will be also limited.

Things are very different if we use appliances of which we know, with good approximation, the force systems they generate. I am thinking mainly to statically determinate mechanics (i.e. cantilevers)^1^ ^,^ ^3^ ^,^ ^4^ ^,^ ^5^, but this concept can also be applied, with less precision, to alpha/beta springs, rectangular and “T” loops. The point is that if we know the force system we apply, then we can predict the dental movement we get, and we have very good chances to do it with good accuracy. Now many orthodontists think that this is only an academic point of view, but on the contrary, is what I do clinically; and to answer to your question: We have developed a software that is helping the clinician in this task. Actually, it is doing also something more useful, in fact, I am not very much interested in predicting the effects of a given appliance, I am mostly interested in designing the mechanical system that will generate the force system leading to the movement I have decided in my treatment planning. So, DMA (Dental Movement Analysis), which is a part of the T3D Occlusogram software - that I use for making a 3d virtual setup -, is able to calculate the force system needed to achieve a specific goal. I design my custom mechanics based on this force system. There is some approximation in the system, depending on possible errors in the estimation of the position of CRes and on the anisotropy of the orthodontic ligament,^18^ ^-^ ^20^ which can make the tissues react differently depending on the force direction. However, I can say after many years using this approach, that my clinical capabilities of designing the optimal mechanics for a given goal are quite good.

**3) The mandibular repositioning is presented by you as an optional treatment for Class II malocclusions with moderate mandibular retrognathism, as well as for many cases of skeletal asymmetry. Can you determine, prior to the repositioning itself, if a specific case is going to positively respond to this approach? Wislei de Oliveira**

I have been doing controlled mandibular repositioning in the last ten years, in patients out of growth and without any TMD symptoms. I remark the word “controlled” because I believe that many repositionings happen during the orthodontic treatment with little control by the orthodontist, who sometimes is not even aware of that. In these cases, the clinician usually overestimates the dental movement role within the treatment results.

The general idea is that the condyle has not a single position in the fossa, corresponding to dental maximum intercuspation, where the patient has good function and no symptoms. I believe that there is often a range of 2/3 mm where we can work safely and I have seen many patients with one or two condyles that in a CBCT are shown off the fossa center, both posteriorly or anteriorly, with no sign at all of TMD. Patients stay in this position because occlusion leads them there and muscles are adapted.

I started to do repositioning in asymptomatic patients after having done it for years (and of course I still do it) in patients with TMD who had lost the correct condyle/disk relation and/or had joint pain. In these patients we often need a controlled advancement of the condyle to recapture the disk or to reduce the pain. Some years ago, with the contribution of Dr. Paola Merlo, who did a master thesis at the University of Siena about this topic,^21^ we started a clinical research project to test the idea of moving the condyle(s) as an alternative to surgery in the treatment of skeletal discrepancies in adult patients with no TMD.^7^

After several years of experience of this approach, I can say that repositioning can be used in many Class II and asymmetric cases where a condylar advancement is possible.

The requisites for mandibular repositioning are that:

1) There is space in the fossa for the condyle displacement.
2) The patient’s neuro-muscular system adapts to the new position.
3) The orthodontist is capable of modifying occlusion to stabilize the new position.

For what concerns the space in the fossa, you should consider that a unilateral condylar advancement of 3 mm provokes a mandibular rotation that corrects the chin and lower incisor transversal position of about 4-5 mm. This covers the great majority of transversal skeletal asymmetries. While with the same bilateral advancement we can only treat completely a moderate skeletal Class II. So, if I see a patient with a severe chin deficiency, I would not recommend this approach as a first choice. 

**Figure 1:**
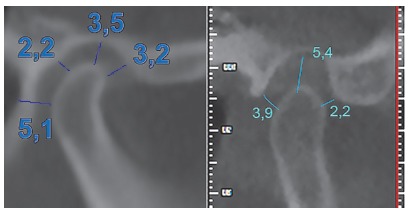
To the left, this condyle allows about 3 mm of advancement. The condyle to the right has much less advancement possibilities. Both the condylar position and the fossa anatomy should be considered.

In all cases where we think in a mandibular repositioning, we need to do a thorough clinical evaluation of TMJ conditions and we need CBCT images of the joint to evaluate if there is space to advance the condyle (Fig 1).

Regarding muscular adaptation, the only thing we can do is a clinical test, for a duration of several weeks. We create an artificial occlusion, using Triad gel bonded mostly to the buccal lower cusps, from the first premolar to the last molars bilaterally, and we see how well the patients adapt to it. This material is quite brittle, so if the patient does not bite consistently in full intercuspation, but has some precontacts, he will break it. So, we leave the patients to bite at least 2 months (at the beginning was even 3 or 4) on this material. We expect that the patient tells us to be comfortable and that he has almost forgotten to have the material in the mouth and of course that no breakage happened in the test period.

I would say that the great majority of the patients, as we have reported in our paper^7^, adapt to the new position. In our first cases (those included in our research), repositioning was done without a previous CBCT control and we had at least ⅔ of adaptations. Now that we filter the cases with the CBCT images, and we exclude immediately patients where the condyles are already too anterior in the fossa, the adaptation rate is surely higher.

The third point is related to the patient initial malocclusion and to the orthodontist capabilities. Class II corrections are definitively simpler to stabilize, while asymmetries often require complex movements and the use of TADs for asymmetric decompensation. In all cases, the orthodontic treatment itself is the real core and difficult part of the therapy.

All in all, I would say that repositioning of the mandible has been one of the major changes in my work in the last 10 years, and I invite the readers to see the paper with the report of the clinical research by me, Dr. Merlo, Dr. Dalsra and Prof. Melsen.^7^

**4) Patients with a Class II malocclusion, mandibular retrognathism, and hyperdivergency are considered critical cases especially if treated with inter-arch force systems to correct the sagittal discrepancy. Could mandibular repositioning be considered a possible alternative to treat such cases? Wislei de Oliveira**

I believe so. Mandibular repositioning can be combined with posterior intrusion done with TADs or any other skeletal anchorage type, resulting in an even more significant change of the chin position. In other words, you can imagine that the posterior intrusion generates an anterior rotation of the mandible and of the condyles, to which you can add an anterior translation obtained by repositioning. Please note that the anterior advancement with Triad gel should always follow the posterior intrusion, to take advantage of a different and more horizontal orientation of the occlusal plane. This is the same concept that Prof. Sato teaches: he uses the multiloop system with intermaxillary anterior elastics to get the Class II correction and hyperdivergency improvement. Now this is often combined with the use of TADs^7^ ^,^ ^8^ ^,^ ^9^.

**5) What is the mean treatment time for mandibular repositioning in an adult patient with Class II and asymmetric malocclusions? What are the biologic responses in the condylar and alveolar area when using this approach in these patients? Wislei de Oliveira**

Sincerely, I have not exact data to answer to this question. However, the variations in treatment duration are very large and depend, besides the patient’s biological response, from the needed dental displacements. These can be minimal or very large. Generally, I have the impression that Class II treatments are shorter, because most of the time you need only to move the front teeth to align and correct the inclination with subsequent extrusion (usually minimal) of the posterior teeth. My impression is that the average duration of these treatments is around 18 months. Asymmetries are often more complicated and often require large transversal and vertical asymmetric movements of the posterior teeth, almost always requiring skeletal anchorage. These treatments are therefore generally longer, and I suppose that the average is around 2.5 years. Anyway, as I said, variations are huge, and I remember an asymmetric case of a woman, to whom other colleagues had recommended an ortho-surgical approach, that I have treated in 4 months with excellent results. This was because I had to move only a couple of teeth to guarantee a proper canine guidance, while all the rest of the work was done by the prosthodontist who had to modify all its prosthetic work on implants.

**6) Do you prefer to activate your transpalatal arches (TPA) and lingual arches (LA) with a statically indeterminate or statically determinate approach? Renato Parsekian Martins**

I work using both ways.

I find the statically indeterminate way of activation more convenient for a series of reasons, mainly this appliance can usually achieve the needed results with a minimal effort by an expert operator and discomfort for the patient. This potential is even larger if we combine the activation of the lingual arches with TADs. However, a proper activation might not be easy to achieve, and mistakes are possible. 

On the other hand, statically determinate TPA and LA provide a known force system with a qualitative constancy (M and F direction are constant and so is the value of M/F), this is of course very important for the biological tissue reaction. It means that the same population of cells will stay for a long period in a specific periodontal and bone region. Errors can be easily avoided with statically determinate appliances with some attention and the use of a force gauge to measure the force produced. The drawback is that sometimes the appliance can take some more time to be constructed and generate more discomfort for the patient. This can be the case, for example, if we need to build up extensions (wrongly called power arms) to reach a specific line of action.

So, I can’t give a definitive answer to your question, nor I can give a general recommendation on this issue.

**7) There is a great tendency on the treatment planning with software as Clincheck, Elemetrix and Dolphin, among others. Most orthodontists are unaware of the digital occlusogram. In your opinion what is the big differential of this tool? Mauricio Sakima**

The digital occlusogram is a software that I have developed at the beginning (1999) because it took too long time to me to execute the manual procedure as I had learned in the Aarhus school^10^. From that time on, I went on improving it without having any commercial intent. I had just the idea to develop a useful tool for me and my students. After many years, it is now a fully developed and mature software, which has some characteristic that are maybe different from the others you have quoted. When I execute an occlusogram, I start to think about those decisions that determine the orthodontic treatment plan: 1) the skeletal facial relation (if I am planning to modify it by surgery, repositioning or growth, or keep it as it is); 2) the symmetry axis of the dental arches; 3) the needed vertical and sagittal desired position of the front teeth, as we see it in the lateral headfilm; 4) the arches shape and size; 5) finally, the maintenance or modification of the dental size (this includes extraction, interproximal reduction, dental build-up, placement of implants).

These are all decision that are taken by the orthodontist routinely. The occlusogram uses these parameters to visualize the treatment planning outcome. In the past, the treatment outcome was represented in 2D; more recently, it can also be analyzed in 3D if the vertical posterior height, the curve of Spee and the cant of the anterior teeth are also decided. At this point everything is automatically displayed in digital models where we can see a virtual setup and the needed movements of the teeth in 3D. I think what most distinguishes the occlusogram from the other software is the integration with DMA software that allows to analyze the movement and find the needed force system for them. This is the first step for a force-driven mechanics design. We have still to work to improve many functions, but I think this is and will be a very interesting feature of our occlusogram.

**8) You have written an article about the correction of the dental midline with the segmented arch approach. Can you describe to us this two-vector mechanics and why do you prefer this technique? Gidalti Bueno Linhares**

The story of the midline correction with a two-vector mechanics might be quite interesting. When I was back from my training in Denmark in 1989, I started to apply what Prof. Melsen had taught to me to move the front teeth transversally as a group, by translation: *“Make a rigid unit and raise from it an apical extension to reach the CRes level. Then apply a transversal force with a cantilever.”* Once back to my practice I tried this, and in the first two or three cases, I was always getting some inclination of the front teeth with the cant of the anterior occlusal plane: this was because the apical extensions my patients could accept were not enough to reach the CRes level.

So, I was disappointed by the system and every time I had to correct the problem adding an intrusive cantilever on the opposite side. Once I decided to apply the intrusion cantilever together with the other one and I was very excited to see that I could get a translatory movement with no cant right away. At that time, in the early ’90, Prof. Burstone in his lectures was teaching that it was not possible to move the front teeth transversally if the apexes had to be moved with the crowns. So, you can imagine how excited I was to have found this new solution. Birte Melsen and I at first wrote in our electronic book that a translation could be achieved if the needed movement was both transversal and intrusive. This was indeed a two-vector mechanics, but at that time, I and Prof. Melsen did not have a real control on how to design these two-vector systems to solve different problems. I went on thinking on vectors and on the possibility of finding a general solution to build up any given force vector using two vectors, and in the year 2000, I have published, together with Prof. Melsen, the paper “Two-vector mechanics” ^11^. With the mathematical procedure described there, you can select two points at your will and calculate two vectors applied to them that summed together will result in any given force system. A small paper, I would say, but I am still very proud of this idea. I think I have designed hundreds of different mechanics based on this mathematics, and I assume that other orthodontists might have done it as well.

As you can see, there is no apical extension and no intrusion component while we have a force passing through the CRes, thus determining a translation.

Sometimes during my courses, I still find people quite surprised to see that I don’t use an apical extension in such a case. Well, I have to admit that my paper is probably not so popular. However, one of the most important things is that the system can be easily tailored to any different condition: if you need a vertical component with any angulation, or to move the apex more than the crown with different centers of rotation (just to keep the problem of the midline correction in a simple 2D representation), you can always easily calculate the perfect two-vector system to correct it.

I refer you to read a recently published case report^12^, in which I showed a case where I needed to apply a force above the CRes. As a matter of fact, I believe you can always design the almost perfect mechanics using the two-vector concept, that’s why I use it quite often and I am very happy to teach it to my students.

**9) Do you think orthodontists can produce better results today than at the beginning of the 80’s when we did not have skeletal anchorage, virtual treatment planning or superelastic wires? Renato Parsekian Martins**

Of course. I started to practice in the early 80s and things are completely different today. I would say that what you mentioned gave the orthodontists the potential to improve the quality and enlarge the possibilities of their therapies. However, I am afraid of one message that often comes along with these instruments, particularly the superelastic wires: *“Everything is possible, everything is easy”*. Some of our colleagues are fascinated by the idea of using something very easy to learn that magically will solve all their problems. But this is only a fantasy. The orthodontist still needs a great amount of knowledge and skill to achieve the best results. I have seen many problems created just by the use of a superelastic wire. In fact, I believe that the outcome of an alignment mechanics performed using a continuous superelastic archwire is not always predictable, and in all cases is never so accurately predictable as it can be if a segmented arch approach is adopted. A few years ago, I have done a test, using the Facebook page of the Biomechanics Summer School (facebook.com/OrthodonticBiomechanicsSummerSchool, which I invite all of our readers to visit) to test the capabilities of predicting dental movements when a straight wire is used. I repeat this test often during my biomechanics courses. So, I invite you to do this test here (Fig 6). You can see the clinical results of this appliance in Figure 12.

**Figure 2:**
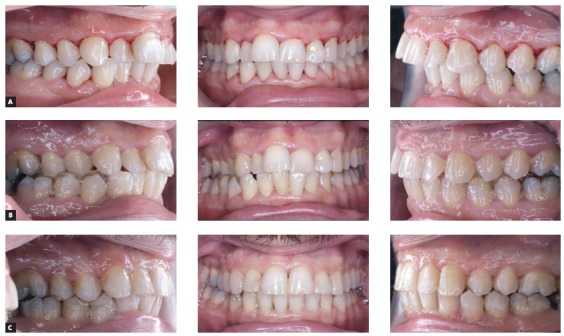
A mandibular repositioning treatment in an asymmetry case. A) Before treatment. B) Repositioning with Triad gel done before the fixed orthodontic appliance treatment. The patient stayed almost 3 months with this occlusion without any further treatment, to verify functional adaptation to the new occlusion. C) After orthodontic therapy with fixed appliance. A small amount of Triad gel was still bonded to teeth #43 and #44 to increase occlusal stability, and would be removed a few months later. A moderate gengivectomy was performed to improve upper front teeth esthetics.

**Figure 3:**
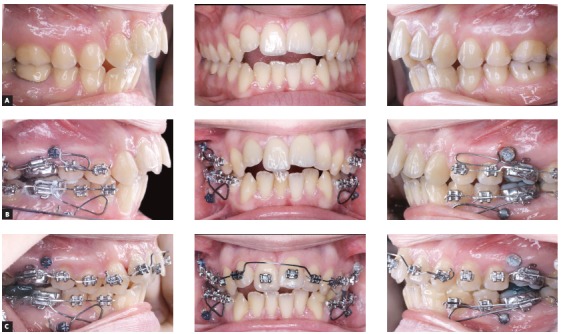
In A) a Class II open bite before treatment. B) After one year of treatment with posterior intrusion, both the vertical and sagittal problems have been partly solved. C) An anterior repositioning with Triad gel completed the correction of canine and molar relations.

**Figure 4:**
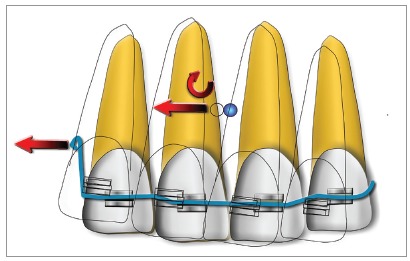
If the apical extension does not reach the CRes level, a certain amount of rotation of the front teeth will take place, resulting in a cant of the anterior occlusal plane.

**Figure 5:**
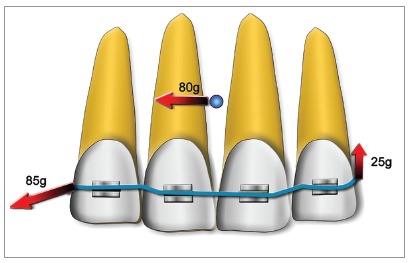
In the diagram, you can see a typical two-vector mechanics for midline correction by translation.

**Figure 6:**
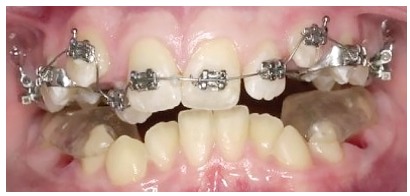
A 0.014-in NiTi archwire is engaged into the brackets of these severely maligned upper teeth. For the following teeth, predict the movement for the next month (circle the movement you predict), then go to Figure 12 to see the actual clinical effects: > Tooth #13: Extrude/Intrude - Buccal crown tip/Lingual crown tip. > Tooth #12: Extrude/Intrude - Buccal crown tip/Lingual crown tip.

Actually, I have seen that in this specific case the average orthodontist, who unfortunately does not have a very large biomechanics knowledge, has quite poor prediction capabilities, and I have to admit that even for myself, such predictions are sometimes not easy at all to perform.

My final message here is that probably we now drive “a more powerful and faster car”, so we can go very far and often with very little effort, but sometimes accidents happen, and the crash can be devastating both for the patient and the orthodontist; so orthodontic drivers should be careful, respect the limits given by their capabilities and the biology and most important of all, read the instruction manuals.

**10) What is your preference regarding skeletal anchorage as auxiliary to mechanics? Plates or screws? Direct or indirect anchorage? Renato Parsekian Martins**

I routinely use TADs placed between the roots, applying both direct and indirect anchorage. Less frequently in the palate or in the buccal shelf. I have used plates a few times when I did not have other options. Screws placed between the roots easily allow an indirect use by connecting a small rigid wire between the TAD head and one tooth of the anchorage dental unit. 

But many times, you can find a way to apply them along the line of action of the force needed to move the active unit. In this way, I can easily build a “perfect” appliance. It is important to place the TAD along this line so that we can apply to the screw a force without applying a moment, since we know that the moment might cause loss of stability in the anchorage screws if they are not osseointegrated and if the moment is acting in a plane perpendicular to the screw axis. You can also use our DMA software to draw the line of action of the needed force and see where it may be convenient to place the screw.

**Figure 7:**
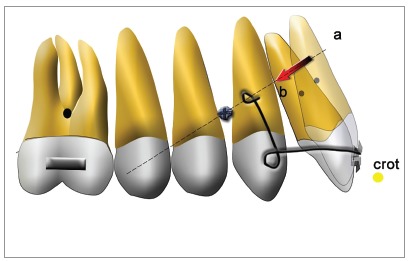
Line of action calculated by the DMA software.

In the example of Figure 7, you can see the line of action of the needed force to displace the incisor from ‘a’ to ‘b’. A TAD can be placed between the roots of the canine and the first premolar, where a cantilever with configuration can be attached. A lot of these examples can be found in Prof. Melsen book about skeletal anchorage.^13^

Regarding screws in the palate, I have been using them as direct anchorage only when the biomechanics was calling for it as the best option. I find more difficult to work with them, without having some specific auxiliary devices, as those designed by Dr. Benedict Wilmes^14^ ^,^ ^15^.

Miniplates are obviously a great anchorage tool; however, since they require a real surgery, I am limiting them to very special cases: until now, I needed to use the Bollard™ anchors, designed by Dr. Hugo De Clerck^16^, only in a couple of cases.

**11) Mastering biomechanics brings predictability to the orthodontic treatment. In which cases you consider that this knowledge is essential? Mauricio Sakima**

There are many factors that I could quote for this answer: age, periodontal conditions, asymmetries, extreme low or high vertical patterns, inherent inconsistency of standard straight-wire systems. All of these are conditions that require a good biomechanics knowledge. But I feel that probably the most important is the amount of dental movement that is planned. I always say to my students “the larger is the dental movement you plan, the most accurate must be your mechanics design”. In small movements, as in finishing stages, the biomechanics knowledge of the clinician usually plays a minor role, and the orthodontist can rely mostly in “shape-driven” mechanics, which is guided by the visual perception of the bracket and wire position. This becomes mostly a matter of personal skill and patience, so sometimes I hear colleagues that talk about the “art of finishing” and I would say that I agree on that and that personally I don’t think I am a Michelangelo of orthodontics. But when the distance to be covered by the teeth becomes larger, then the visual aid and the talent becomes less important and the knowledge of biomechanics, maybe a more “scientific” side of our profession, plays a larger role.

**Figure 8:**
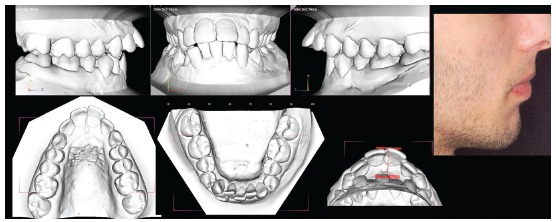
Example of the use of T3DO and DMA to plan and design the mechanics.

I will give you a clinical example to let you understand this concept. The case in Figure 8 had a huge overjet of more than 11mm, although the molar and canine relations were not far from a Class I, and the profile showed only a moderate Class II pattern, not being a concern for the patient. Teeth #11 and #21 were injured in the past and it was decided to extract them together with tooth #42, which had periodontal problems, and tooth #34.

**Figure 9:**
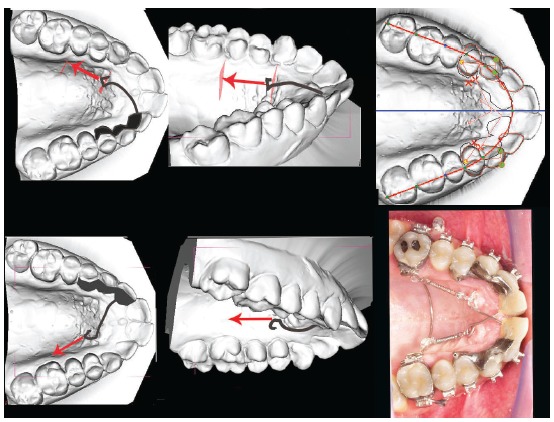
Occlusogram and mechanics design in a case where extraction of the two upper central incisors would be done. The red vectors represent in 3D the required force for the movement planned with the occlusogram. These forces are calculated by the DMA software, which is part of our biomechanics design system. The planned movements are rotations of two groups of teeth, with the center of rotation being represented by the yellow area in the occlusogram.

**Figure 10:**
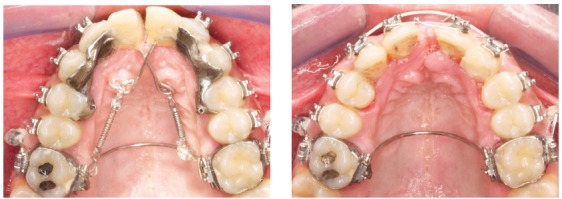
Upper arch shape at start and after seven months from the application of the designed mechanics. Later, a few months were needed to perform movement of single dental units.

**Figure 11:**
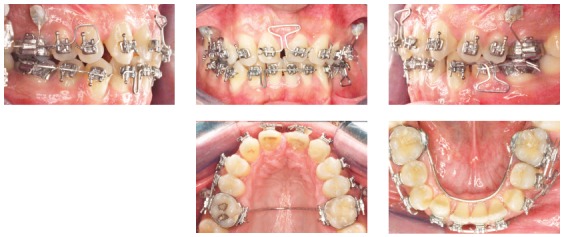
Ten months of treatment, before the shape modification of anterior teeth. In the lower arch, teeth #42 and #34 were also extracted. Overjet and overbite are now normal.

**Figure 12:**
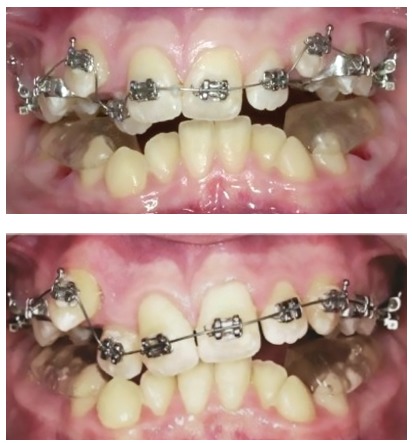
This is the result of the mechanics of which I asked to predict the effect (Fig 6). Were you able to do a good prediction?

The T3DO and the DMA software were used to plan the needed dental movements and design the mechanics, as it is shown in Figure 8. As you see, although brackets were placed at start of treatment, the mechanics is based on a structure fabricated to orientate the coil spring along the needed line of action for the movement of teeth #14, #13 and #12 on one side, and of teeth #24, #23 and #22 on the other side. The space to be closed between the two lateral incisors was about 16mm. The orthodontic therapy was based in this case on individual mechanics analysis and design, and the results that we have obtained in a relatively short time confirm the efficiency of such approach when large displacements are needed.

**12) You have organized and developed a biomechanics summer school that trains orthodontists from all over the world every year during the summer in north hemisphere. What are the main complains and difficulties that you observe every year when the students begin the course, and are these difficulties an effect of the lack of biomechanics knowledge during their formation as orthodontists? Gidalti Bueno Linhares**

Generally, the students have a quite hard impact with our teaching. Although they generally have a high motivation to attend a five weeks course abroad, most of them do not expect that our approach to orthodontics is so different from standards. During the course they must learn how to do occlusograms to set the goal in 3D, to calculate the needed force system and amount of anchorage, and sometimes they must do calculations to design the optimal appliance. All this is biomechanics, not talent or magic. It can be taught and learned, and I believe that this is a factor that sets a difference among orthodontists. I don’t believe that someone can be a good orthodontist without a good knowledge of biomechanics. And this knowledge unfortunately is not often given in postgraduate university courses around the world. At the end of the whole program I often hear this question: *“Why they didn’t teach this to me in the university?”.* This problem is regarding the whole globe, and I would say that in the average in Brazil, the biomechanics knowledge is better than in many other places, but still not enough.

My mission for the rest of my life will be teaching biomechanics and prepare others to teach it. With this in mind, we have founded an association called “Biomede”, including people that are interested in teaching Biomechanics. We will have the first world symposium of orthodontic biomechanics next January 2019 in Qatar, with many of the greatest experts in the field speaking. We hope that this will be another opportunity to promote a different orthodontic thinking and clinical approach. You are all invited to attend (www.biomede.org). Another news for you is that some members of Biomede are planning to make a South American edition of our European summer course, in which I might be an invited speaker, so it will be not so difficult for the doctors in this continent to have access to this formative program.

**13) In your opinion, why is orthodontic biomechanics in the background in orthodontic programs and meetings? Gidalti Bueno Linhares**

This is a sad truth for me. On top of this, many colleagues are wrongly convinced that biomechanics knowledge is not necessary at all. Of course, as you can imagine this idea is well seeded among those who have never studied biomechanics in depth. I don’t know on which basis they are convinced about that. This would be a reasonable conviction if they never experienced a failure, but this is not very likely. I do believe that many orthodontists have some difficulties in treating a significant percentage of their cases and that this is probably related to a lack of knowledge of orthodontic biomechanics. I do a lot of retreatments in my practice, maybe 10% of the cases, and all of these are patients who were treated with straight wire technique. In these cases, the clinician has lost control of the dental movement, has no understanding of the reason for that and, obviously, has no solution for it.

Maybe these colleagues are honestly convinced that these treatments are doomed to failure in any case, but this is not true. In fact, I would say that I can retreat most, if not all, of them with a reasonable success. 

I believe that there are two key factors that contribute to the conviction that biomechanics is not really useful for the clinical practice. First of all, the commercial companies and also some gurus have often aimed to sell materials and methods as easy solutions for every case: *“Buy my bracket/wire/prescription/device and everything will be fine.”* This gives the illusion that you can practice without a proper training. Secondly, I believe that the amount of biomechanics knowledge that an orthodontist needs to know to make it clinically useful is quite a lot. The few concepts that are taught in most postgraduate courses are not enough to allow any practical use of it by the orthodontist; therefore, the students are going to forget about them soon once out of the school.

The problem is also that the least widespread is this knowledge, the less teachers will be available in schools and speakers in meetings.

In fact, I believe that the lack of biomechanics teaching is mainly due to the fact that there are too few experts around the world capable of transmitting this knowledge. I hope that our Biomede association will be able to increase this number of valid teachers in the future.

**14) We know that you have released an e-book in 1992, and you have just finished an exercise book. Why you decided 26 years ago to make a digital book and not a print version and how you keep this book up-to-date? Gidalti Bueno Linhares**

After my period in Denmark in 1990, I started to think how to organize the great amount of information I had received during that period and what I had been reading in many papers about biomechanics. I had just bought my first PC and was eager to find a way to exploit the new “toy” (at that time, its cost was probably corresponding to my salary of a couple of months, so quite an investment). I have read at that time some books by Ted Nelson and other authors about Hypertext^17^. This was the ground for the development of the world wide web concept and the HTML language, which was released in 1993. I was fascinated by the idea of having a book that could be read without following a linear path, but “jumping” between different pages or concepts. All this seems very natural now. We are all used to “navigate” the web. At those times, there was no web and a few visionaries were developing specific software to produce these hypertexts. So, I started with the idea of producing a “Biomechanics Hypertext”. By the way, when I started the project I only thought of the text, then I have realized that technology was allowing to add simple drawings with minimal resolution and 16 colors. There was the problem of memory support. My hard disk was 20 Mb and I needed external optical devices to store the images (after a while, we got 256 colors) when we could include very low-quality photographic images. In 1991, I had done the initial backbone of the project (programming 90% of it on my own and asking some help to other programmers, for the most difficult parts) and I showed it to Birte. I needed her cases to complete the “book”. She was not very much convinced at the beginning so she said I should complete the book on my own before she decided whether to be part of the project. I have been working almost in every spare moment to this project, and I finally convinced her to give me some of her clinical material and to review the text. In October 1992, we presented “La Biomeccanica in Ortodonzia” in a CD-ROM. It was a big novelty and my colleagues were buying an external CD-ROM reader in order to see the software, since most machines didn’t have it.

It was a huge success in Italy, so I decided to create an English version 1.0, which was published in 1994. Since then, we have modified many things in the structure and always add contents. We had reviews in many orthodontic international journals and in the mid-90s, I was often invited to speak at meetings about the book, not about biomechanics. In 2013 the actual v. 4 was released, which is only web-based and has continuous updates.

Sometimes I come back from my office with a nice clinical picture, or I have prepared some slides or videos for lecturing, and I am adding them to the “book”. Very often people ask me *“Why don’t you make a printed book”*, and I answer: *“There are many of them, this is different.”* So, if you want to read a hypertext in biomechanics this is the only product available. 

You asked me about the exercise book, at which I am still working. Students in my courses ask me more and more exercise to test their capabilities. For a long time, I was just making a drawing during the lecture at the board and they had to work at it. Now I am collecting these exercises, and they will be printed on paper!

**15) Can you tell us how living together with Birte Melsen and Charles Burstone guided your orthodontic life, and how you can contribute to the continuous development of this field (biomechanics) even with this trend of “fast orthodontics”? Gidalti Bueno Linhares**

We are speaking about two real giants in modern orthodontics. I first heard them in a conjunct course they gave in Milan in 1985. At that time, I had only 2 years of experience as an orthodontist. I was fascinated by the fact that they were presenting to me a rational explanation of the many things that happened during the orthodontic treatment. I was also amazed by the cases they were showing. These were indeed beyond my imagination.

After that, I have started to study, reading their papers, and in 1988 I went to Denmark for a 3 months period and I had the great luck to meet Birte Melsen personally. She has taught really many things to me and I have had the honor of having her as a mentor. We have been working together in many projects starting with the multimedia textbook “Biomechanics in Orthodontics”, publishing many papers together, including the last one on mandibular repositioning, I was involved in two books she has edited and now we teach together in the Biomechanics summer school. It’s very easy to say that without Birte Melsen, I would not have achieved most of the results I had in the profession.

Unfortunately, I did not have so frequent personal contacts with Charles Burstone, but my debt with him is huge: I have spent days in studying all his papers and I was also rewarded by his esteem, when he asked me to write a chapter in his last book.

Well I don’t feel comfortable to say which can be my contribution to the field of biomechanics. I am trying to teach and to show a path that brings to an understandable and logical clinical use of biomechanics. I hope that my students appreciate me for the method, for the rational clinical thinking and for the passion I put into teaching, rather than for a single paper or “invention”. This kind of appreciation would be a great reward for me. I am also particularly happy to see some of my students becoming teachers themselves and spreading the knowledge they received from me.
